# Peer support and learning outcomes in “Study With Me” among Generation Z college students: mediating roles of motivation, test anxiety, and self-efficacy

**DOI:** 10.3389/fpsyg.2025.1582857

**Published:** 2025-06-30

**Authors:** Rui Hou, Ling Jin, Jian He, Jin-Liang Wang

**Affiliations:** ^1^Boya College, Ningxia University, Zhongwei, Ningxia, China; ^2^College of Innovation and Entrepreneurship, Shandong Huayu University of Technology, Dezhou, Shandong, China; ^3^Qinxue College, Ningxia University, Yinchuan, Ningxia, China; ^4^Center for Mental Health Education, School of Psychology, Southwest University, Chongqing, China

**Keywords:** Study With Me, Generation Z College Students, peer support, learning outcomes, learning motivation, test anxiety, self-efficacy

## Abstract

**Background:**

“Study With Me (SWM),” a popular trend among Generation Z college students, enabling self-directed learning through livestreams or prerecorded videos and offering peer support in virtual settings. However, its impact on learning outcomes remains underexplored.

**Purpose:**

This study examines how peer support impacts learning satisfaction and engagement in SWM, focusing on intrinsic motivation, extrinsic motivation, test anxiety, and self-efficacy as mediators, comparing livestreams and prerecorded formats.

**Methods:**

Data were collected from 509 Chinese college students who had engaged in SWM on Bilibili for at least three consecutive months. Structural equation modeling (SEM) and multigroup analyses were conducted using SPSS 30.0 and AMOS 26.0.

**Results:**

Peer support positively predicted intrinsic motivation, extrinsic motivation, self-efficacy, learning satisfaction, and engagement, and negatively predicted test anxiety. Intrinsic motivation, test anxiety, and self-efficacy mediated the effect of peer support on learning satisfaction, while intrinsic motivation, extrinsic motivation, and self-efficacy mediated its effect on learning engagement. Multigroup analysis revealed stronger peer support paths among livestream learners and stronger self-efficacy paths among prerecorded video learners.

**Conclusion:**

These findings underscore the importance of peer support in fulfilling psychological needs and improving learning outcomes in SWM, offering practical implications for enhancing digital learning and supportive online environments.

## Introduction

In recent years, “Study With Me (SWM)” has become popular among Generation Z college students. This method of recording, watching study process videos or study in livestreams software with others is known as SWM (Wang and Zhang, 2021). In 2024, more than 14.7 million people subscribed to “SWM YouTuber” YouTube videos made by the content creator “Lofi Girl,” (YouTube, 2024) and the maximum number of views for a single video on this channel was more than 700 million. In China, on the website “Bibibili” 10,293 live SWM rooms (Bilibili, 2021) are hosted daily, attracting more than 327 million participants (Blue Lion Inquiry, 2023), more than half of whom are college students. SWM has come to represent a type of learning and social culture for Generation Z.

The reason for this situation could be that members of Generation Z (1997–2012), who were raised during a period of rapid progress in internet and mobile technology, favor digital tools and online resources for socializing and learning. Compared to previous generations, Generation Z college students demonstrate a stronger pursuit of personal privacy and freedom of choice. Moreover, they also exhibit desires for social support and interpersonal interaction (Hong, 2023). Students engaged in self-directed learning become more reliant on opportunities that allow them to negotiate learning in a personalized manner. This makes the learning experience more engaging and meaningful (Younas et al., 2024). Study With Me is an online self-directed learning practice developed by college students in the digital era to combine solitary study with peer interaction. Compared to traditional self-directed learning environment, SWM have significant advantages due to its interactivity and accessibility nature. However, challenges such as susceptibility to distractions and irregular study engagement remain, potentially compromising the effectiveness of SWM. Learning satisfaction, which measures learners’ perceptions of their learning outcomes, is a key indicator of learning outcomes (Shin, 2003). Similarly, learning engagement-referring to the time and effort students invest in effective learning practices, reflects their current academic status and profoundly influences academic outcomes (Li and Huang, 2010).

Multiple studies have highlighted the crucial role of peer support in enhancing learning satisfaction and learning engagement in SWM. Zhu and Shi (2021) discovered that peer companionship significantly increased learners’ enthusiasm for online learning, Lee et al. (2021) contended that it can prolong the duration and satisfaction of learning. In SWM, peer support enables the co-construction and sharing of educational experiences, facilitating the exchange emotional experiences and learning strategies, which extends study time and improving efficiency (Lee et al., 2021). Moreover, peer interactions can enhance learning motivation, alleviate test anxiety and reduce burnout (Hou et al., 2023). Despite the recognized benefits of peer support, research examining its specific effects on learning outcomes is scant (Rotar, 2022). This lack of comprehensive evidence has led some scholars to question the educational value of SWM, labeling it as performative rather than true learning. Given the debates surrounding the educational efficacy of SWM and its reliance on peer interactions, a deeper investigation into the actual impact of peer support on learning outcomes in SWM is necessary.

Digital technology has catalyzed the diversification of SWM practices, which now predominantly take the form of livestreams and prerecorded videos. Livestreams facilitate real-time interaction and communication, potentially enhancing learning dynamics and fostering the development of study habits, among students who value peer presence and enjoy engaging in online study communities (García-Morales et al., 2021). In contrast, prerecorded videos incorporate audiovisual aesthetics and self-paced time management, offering personalized and flexible learning experiences that encourage prolonged immersion (Dumford and Miller, 2018). This diversification highlights the potential of SWM to accommodate varied learner preferences. However, empirical studies comparing the distinct effects of these two formats on learning outcomes remain limited. In particular, how peer support operates across SWM formats in shaping learning satisfaction and learning engagement is still insufficiently understood.

Therefore, the present study aims to: (1) examine the effects of peer support on learning satisfaction and learning engagement among Chinese college students using SWM, focusing on the mediating roles of internal motivation, external motivation, test anxiety, and self-efficacy. (2) It also explores whether these pathways differ between livestreamed and prerecorded formats.

## Literature review

### The impacts of peer support on learning outcomes

In the context of online learning, learning satisfaction (LS) and learning engagement (LE) have been widely recognized as key indicators that can be used to measure learning results (Lane et al., 2021). Peer support is an important factor influencing the improvement of learning outcomes and can help students cope with learning challenges, increase their level of learning engagement and academic performance, and improve their satisfaction by enhancing their mental well-being (Hou et al., 2023). Peer support (PS) refers to the process in which peers provide emotional, evaluative, and informational support to each other, its significant role in traditional learning has been well-documented (Liang, 2008). Researchers have found that in SWM interactions among peers can enhance learning enthusiasm (Zhu and Shi, 2021). Learners collaboratively build their learning environments, share emotional experiences (Lee et al., 2021), and exchange learning strategies, which can extend study time and improve learning efficiency (Hou et al., 2023). Jung and Choi (2023) showed that peer support increases learning engagement and the amount of time invested in learning. Wang and Zhang (2021) discussed the effectiveness of emotional support and information exchange with regard to enhancing learner satisfaction. Wu and Zhang (2021) reported that peer interaction can increase enthusiasm for learning. The fact that SWM has also been found to extend the unique type of support known as “peer supervision” is particularly noteworthy, that is, learning under the gaze of others can result in greater self-discipline (Hou et al., 2023).

### The mediating effects of learning motivation, self-efficacy and test anxiety

Learning motivation is an important factor for learning success and a key issue for students who participate in online learning (Tang et al., 2021). Learning motivation includes both internally and externally generated motivation. Intrinsic motivation (IM) primarily driven by internal desires such as curiosity, learning, or personal satisfaction (Ryan and Deci, 2000). Extrinsic motivation (EM) involves undertaking tasks to earn rewards or avoid punishments from external sources. Both types of learning motivation significantly influence learning strategies and outcomes in educational contexts. Due to different learning motivations and experiences, learning activities and engagement levels differ in SWM (Kim and Ryoo, 2023). Wang et al. (2022) reported that learners care about the gaze and evaluation of others, thus leading to a decrease in learning efficiency. Hong (2023) emphasized the importance of internal motivation, claiming that learners who exhibit high levels of internal motivation are more willing to learn and more persistent.

Research has shown that anxiety concerning tests is an important factor for learners who participate in SWM (Kim and Ryoo, 2023). In a competitive society, test anxiety (TA) is a universal issue. College students faced with test pressure of graduation, further education, employment impacting both their academic performance and mental health (World Health Organization, 2017). Amid a global economic slowdown and decreasing job stability, college students in China show a strong preference for positions within the system (China General Social Survey, 2021). This trend is evident from the increasing applicant-to-position ratio in the National Civil Service Exam, which escalated from about 37:1 in 2021 to approximately 73:1 in 2024 (Xinhua News, 2023). There has been an increase near 1 million candidates for the national postgraduate entrance examination from 2021 to 2024. Wang et al. (2022) suggested that forms of interaction such as silent companionship, bullet screen communication, and voice calls can alleviate anxiety and loneliness among students preparing for exams. Hou et al. (2023) emphasized the relationship between test anxiety and learning engagement and reported that alleviating test anxiety can improve learning efficiency. Facing intense competition, the phenomenon of alleviating mitigate educational involution and test anxiety through SWM learning has become a unique online culture among contemporary Chinese youth (Hong, 2023).

Self-efficacy (SE) reflects a learner’s belief in their ability to accomplish goals through effort when faced with challenges. Learners with high self-efficacy often exhibit increased vigor in challenging situations (Jung and Choi, 2023), and they are particularly more likely to succeed when facing demanding tasks in specific learning environments (Tang et al., 2021). In online learning, learners who exhibit high levels of self-efficacy tend to believe in their own success and are willing to invest more effort (Park and Yun, 2018). In SWM, positive feedback from peers boosts learners’ confidence in their capabilities, displaying greater self-control than usual when in front of their peers. These learners actively participate in learning, effectively manage their time and energy, improve their self-discipline, and are more likely to achieve their learning goals.

### Study With Me types: livestreams and prerecorded videos

The Development of digital technology and the influence of COVID-19 Epidemic, has made online learning popular all over the world (Younas et al., 2022), also let SWM has been seen and applied by more learners. SWM can generally be divided into live streams and prerecorded videos.

Brown and Wade (2020) posited that live streams enhance learners’ sense of authenticity and participation and that the learning experience highly akin to in-person learning. Live stream learners employ webcams or smartphone cameras to broadcast their studying live, enabling real-time interaction with peers through both voice and text. To emulate an scene akin to a library or study hall, learners voluntarily establish online study groups, by online meeting software (such as Zoom and Tencent Meeting (Desk Mates Cloud Study Room, 2024)) or livestream websites (such as YouTube and Bilibili), they share their study process, collectively fostering a “studying together” atmosphere (Lee et al., 2021). Live stream often typically enforce rules reminiscent of traditional classrooms, such as scheduling study periods of 50 min followed by 10-min breaks, communication is restricted to break times, and learners who do not adhere to these rules may be removed from the session, this interactive setup replicates traditional classroom dynamics. During this process, some learners’ smartphones for broadcasting can inadvertently reduce unnecessary phone usage and enhance study efficiency.

Livestreams require stable internet connections and digital devices. Consequently, the learning environments for livestreams are primarily set in fixed indoor settings, which not fully accommodate the diverse and personalized needs of learners. Conversely, prerecorded videos incorporate various self-directed learning strategies and foster a virtual learning environment conducive to enhancing learner concentration and guiding learners through their educational activities (Wang and Zhang, 2021).

Prerecorded videos allows for post-editing, which means that the filming locations can vary widely—from roadside cafe to forests and beaches-offering learners an array of visually and hearing stimulating environments, a distinctive feature of videos is the inclusion of ASMR elements, characterized by soothing audio-visual stimuli that evoke a relaxing sensation, comfort and pleasure, alleviate stress and anxiety, creating a more enjoyable and effective study experience (Li and Li, 2023). Stojanovic (2023) posits that prerecorded videos (Bao, 2021), often incorporate time management tools such focus (Wang and Zhang, 2021) as the Pomodoro Technique, designed to optimize study rhythms and maintain. Prerecorded videos enrich the learning experience and facilitate a learning atmosphere that learners can choose based on their emotional state, study preferences and needs, proving especially beneficial for those who prefer self-directed learning. Different types of online learning have different effects on learning methods and outcomes (Jiang et al., 2017). While prerecorded videos offer flexibility and personalized, they may lack the real-time interaction found in live streams, which could influence learning satisfaction and learning engagement differently.

### Theoretical framework

This study explores the application of Social Cognitive Theory (SCT) and Social Presence Theory (SPT) in creating effective online learning environments. SCT posits that the unique characteristics encompassing the environment, participants, and their interactions—can significantly influence learning behaviors and outcomes. It emphasizes the importance of self-efficacy, suggesting that learners adjust their learning behaviors based on the observed performances of their peers (Bandura, 1989). Following this logic, in SWM, when learners receive support and encouragement from peers, their self-efficacy is supposed to enhance due to increased peer interaction. Concurrently, SPT highlights the impact of perceiving others’ presence, which significantly influences learners’ emotional states (Short et al., 1976). Within SWM, this perceived social presence from peers fosters encouragement and attention, subsequently enhancing learners’ motivation. Learners engage in various forms of interaction such as video sharing, discussion forums, and real-time chats, which enrich their learning experiences and enhance community engagement. Integrating these two theories, the study proposes an analytical framework for investigating the impact of peer support on learning outcomes within SWM, this provides a more holistic understanding of how peer support can enhance learning engagement and satisfaction in digital learning spaces.

### Statement of the study

**Figure 1:**
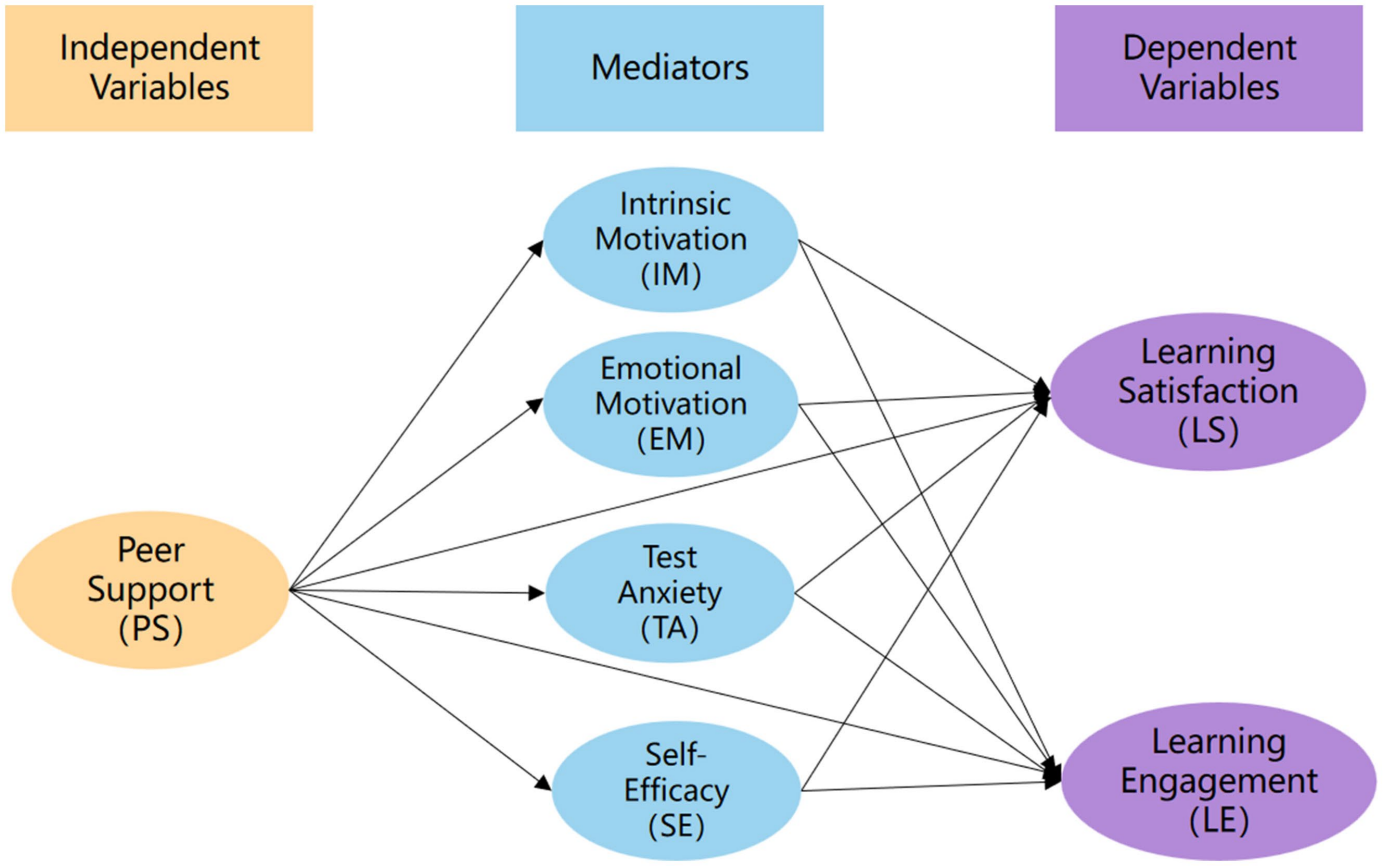
The relationships of the study variables.

Students’ learning satisfaction and learning engagement with online learning are closely associated with their academic performance and are widely used as indicators of digital learning outcomes (Lane et al., 2021). Study With Me (SWM), a new online study format, has gained increasing popularity among Generation Z learners by combining self-directed learning with a sense of social presence and community (Hou et al., 2023). In SWM, peer support is considered a key factor in reducing isolation, improving focus, and extending study time (Wang and Zhang, 2021). Variables such as intrinsic motivation, extrinsic motivation, test anxiety, and self-efficacy are known predictors of learning satisfaction and learning engagement (Younas and Dong, 2022; Tang et al., 2021; Park and Yun, 2018). However, the mediating roles of these variables in the relationship between peer support and learning outcomes in SWM remain unclear and warrant further empirical investigation. Building on previous research, this study proposes a structural model to examine how peer support influences learning satisfaction and engagement through key psychological mediators. It also incorporates a comparative analysis to explore potential differences between livestreams and prerecorded Videos SWM. This study provides empirical evidence for understanding how peer support impacts students’ learning outcomes in SWM. It also offers practical insights for designing more personalized engaging digital study environments. The proposed framework is illustrated in Figure 1.

### Study hypotheses

*H1:* In Study With Me (SWM), peer support (PS) positively predicts students’ intrinsic motivation (IM), extrinsic motivation (EM), self-efficacy (SE), learning satisfaction (LS), learning engagement (LE), while negatively predicting test anxiety (TA).

*H2:* Intrinsic motivation (IM) mediates the relationship between peer support (PS) and both learning satisfaction (LS) and learning engagement (LE) in SWM.

*H3:* Extrinsic motivation (EM) mediates the relationship between peer support (PS) and both learning satisfaction (LS) and learning engagement (LE) in SWM.

*H4:* Test anxiety (TA) mediates the relationship between peer support (PS) and both learning satisfaction (LS) and learning engagement (LE) in SWM.

*H5:* Self-efficacy (SE) mediates the relationship between peer support (PS) and both learning satisfaction (LS) and learning engagement (LE) in SWM.

*H6:* The structural relationships among peer support (PS), mediating psychological variables (IM, EM, TA, SE), and learning outcomes (LS, LE) differ significantly between students who use livestreams and those who use prerecorded SWM videos.

## Research methods

### Study locale and population

This exploratory study was conducted among Chinese college students who regularly engaged with SWM on Bilibili. Bilibili was selected as the research platform due to its large user base of Generation Z users, extensive availability of SWM prerecorded videos, and a dedicated SWM livestreams category featuring real-time interaction and peer engagement. All participants took part voluntarily, and informed consent was obtained prior to data collection. The study protocol was approved by the Ethics Committee of NingXia University, ensuring adherence to ethical standards throughout the research process.

### Sampling

The sample size for this study was *N* = 509, determined through convenience sampling. Recruitment announcements and the questionnaire link were disseminated through multiple online channels in May 2024, including 10 WeChat groups for college students and 10 SWM fan social communities. Participants need to: (1) be currently enrolled undergraduate students, and (2) have engaged in SWM on Bilibili for at least three consecutive months. To ensure demographic diversity, students from different academic years, genders, and academic disciplines were intentionally included. Among the valid responses, 301 participants identified as female and 208 as male. Participants’ academic year distribution was: 155 freshmen (30.5%), 117 sophomores (23%), 110 juniors (21.6%), and 127 seniors (25%). With regard to academic discipline, 327 participants (64.2%) were enrolled in humanities and social sciences, while 182 (35.8%) were pursuing majors in the natural sciences. Regarding SWM usage, 42.4% used live streams and 57.6% used prerecorded videos. Daily SWM engagement time was categorized as less than 1 h (48.1%), 1–3 h (47%), and over 3 h (4.9%).

### Data collection process

A structured questionnaire was distributed through online survey links in May 2024 to collect primary data aligned with the research objectives. The questionnaire incorporated mature scales and was administered via an online platform. All items were rated on a six-point Likert scale ranging from 1 (strongly disagree) to 6 (strongly agree). Data were screened for completeness, and only valid responses were retained for analysis. Anonymity and confidentiality were assured throughout the process, and all data were used solely for academic purposes.

### Strategy of study

Structural equation modeling (SEM) was conducted using SPSS 30.0 and AMOS 26.0. First, descriptive statistics were calculated to summarize participants’ demographics (e.g., gender, major, academic year, average daily time on SWM). Independent sample *t*-tests were used to compare key variables between the livestream and prerecorded video groups. Second, the model was estimated using maximum likelihood estimation (MLE), which offers unbiased and efficient parameter estimates under conditions of multivariate normality and adequate sample size (Hair et al., 2009). Third, confirmatory factor analysis (CFA) was conducted to validate the measurement model comprising seven latent constructs: peer support, intrinsic motivation, extrinsic motivation, test anxiety, self-efficacy, learning satisfaction, and learning engagement. Internal consistency was assessed using Cronbach’s *α*, while composite reliability (CR) and average variance extracted (AVE) ensured construct reliability (Fornell and Larcker, 1981). Discriminant validity was examined via the Heterotrait–Monotrait (HTMT) ratio, with values <0.85 indicating acceptable discriminant validity (Henseler et al., 2015). Fourth, the structural model’s fit was evaluated using multiple indices: χ^2^/*df* (acceptable if ≤3.0), CFI and TLI (≥0.90), RMSEA (≤0.08), and SRMR (≤0.08), following the criteria of Hair et al. (2009). Standardized path coefficients (*β*) were used to interpret direct effects. Fifth, measurement invariance was assessed across four levels (configural, metric, scalar, and residual) before conducting multigroup analysis. Model comparisons relied on chi-square difference tests and ΔCFI, with a change > 0.01 suggesting a significant loss of fit. Finally, multigroup SEM was used to test path differences between livestream and prerecorded groups. An unconstrained model (freely estimated parameters) was compared with a fully constrained model (equal paths across groups) using chi-square difference tests. To identify specific differences, each of the 14 structural paths was tested individually.

### Operationalization of study variables

This study examined seven key variables: one independent variable (peer support), four mediators (internal motivation, external motivation, test anxiety, and self-efficacy), and two dependent variables (learning satisfaction and learning engagement). In addition, the type of SWM usage (livestreams vs. prerecorded videos) was included as a grouping variable to compare potential structural differences between user groups.

### Demographic information

The demographic section collected information on participants’ gender (male and female), academic year (freshman, sophomore, junior, or senior), and major (humanities and social sciences or natural sciences). Participants were also asked to report the type of SWM content they used (live streams or prerecorded video) and their average daily duration of SWM use (less than 1 h, 1–3 h, or more than 3 h).

### Instrument of study variables

This study employed well-established and widely validated instruments, all adapted into Chinese and verified for psychometric reliability. To enhance the parsimony and stability of the structural equation model (SEM), item parceling was employed given the large number of latent constructs and indicators. As recommended by Bandalos (2002), this technique reduces sampling error, improves model fit, and stabilizes parameter estimates in complex models. Each latent variable in this study was represented by three item parcels.

The Peer Support Scale, which was developed by Liang (2008), was used to measure peer support in the respondent’s network and included 7 items (e.g., “Many things I can’t discuss with people around me can be communicated well online”). In our sample, items were grouped into three parcels (PS1–PS3), with Cronbach’s *α* values of 0.848, 0.839, and 0.822, respectively.

The Learning Motivation Scale, which was developed by Amabile et al. (1994) and revised by Yu et al. (2017), comprising 17 items divided into intrinsic motivation (e.g., “The more challenging the problem is, the more eager I am to try solving it”) and extrinsic motivation (e.g., “One of my primary motivations for study hard is the desire to gain recognition and appreciation from others”). Items were parceled into three parcels: Cronbach’s *α* values were 0.896, 0.875, and 0.890 for intrinsic motivation (IM1–IM3), and 0.792, 0.785, and 0.767 for extrinsic motivation (EM1–EM3).

The Test Anxiety Scale, which was developed by Driscoll (2007), included 10 items (e.g., “I worry so much before a major exam that I am too worn out to do my best on the exam”). In this study, items were parceled into three parcels (TA1–TA3), with Cronbach’s *α* values of 0.888, 0.821, and 0.885.

The Self-Efficacy Scale, which was developed by Schwarzer and Jerusalem (1995) as well as Wang et al. (2001), included 10 items (e.g., “I can always manage to solve difficult problems if I try hard enough”). In this study, items were parceled into three parcels (SE1–SE3), with Cronbach’s α values of 0.923, 0.923, and 0.873.

The Learning Satisfaction Scale, which was developed by Shin (2003), was used to investigate student satisfaction with online learning experiences. We consulted three experts specializing in educational technology and educational psychology, based on the suggestions made by these experts, we revised this measure to include a total of 6 items (e.g., “Study with Me is a valuable experience to me”). In our sample, items were grouped into three parcels (LS1–LS3) with Cronbach’s α values of 0.938, 0.932, 0.951.

The Learning Engagement Scale, which was developed by Schaufeli et al. (2002) and revised by Li and Huang (2010), was used to measure student engagement in learning. The scale included three dimensions—dedication (LE1), vitality (LE2), and focus (LE3)—featuring 17 items (e.g., “When I study, I am full of energy”). In our study, with Cronbach’s *α* values of 0.958, 0.947, and 0.938, respectively.

## Results

### Descriptive statistics

Table 1 presents the means and standard deviations of the seven psychological constructs across the livestream group, prerecorded video group, and the overall sample. The average scores across all participants ranged from 2.988 to 4.295, with standard deviations for each variable remaining below 1.00. Overall, the livestream group reported higher mean values than the prerecorded group across all variables, except for test anxiety. Independent samples *t-*tests revealed significant differences between the two groups in peer support (*t* = 6.848, *p* < 0.001), intrinsic motivation (*t* = 2.336, *p* < 0.05), emotional motivation (*t* = 2.518, *p* < 0.05), self-efficacy (*t* = 3.710, *p* < 0.001), learning satisfaction (*t* = 2.985, *p* < 0.01), and learning engagement (*t* = 2.695, *p* < 0.01). No significant group difference was found for test anxiety (*t* = −1.360, *p* > 0.05).

**Table 1:** Descriptive statistics.

Variables	Livestreams (*N* = 216) M/SD	Prerecorded videos (*N* = 293) M/SD	Overall (*N* = 509) M/SD	*t*
PS	4.328/0.720	3.885/0.721	4.073/0.753	6.848***
IM	4.401/0.896	4.217/0.864	4.295/0.882	2.336*
EM	4.290/0.804	4.111/0.785	4.187/0.797	2.518*
TA	2.921/0.965	3.038/0.961	2.988/0.963	−1.360
SE	4.376/0.767	4.107/0.834	4.221/0.816	3.710***
LS	4.492/0.936	4.232/0.992	4.342/0.977	2.985**
LE	4.373/0.748	4.189/0.774	4.267/0.768	2.695***

PS, Peer support; IM, Internal Motivation; EM, External Motivation; TA, Test Anxiety; SE, Self-Efficacy; LS, Learning Satisfaction; LE, Learning Engagement. *p < 0.05, **p < 0.01, ***p < 0.001. Overall represents the combined descriptive statistics for the full sample.

### Results of the measurement model

The confirmatory factor analysis (CFA) results demonstrated an acceptable model fit for the full measurement model: *X2* = 397.468, *df* = 168, *X2/df* = 2.37, CFI = 0.978, TLI = 0.973, RMSEA = 0.052, SRMR = 0.034. Regarding convergent validity, all standardized factor loadings ranged from 0.761 to 0.955, exceeding the recommended threshold of 0.50 (Hair et al., 2009). Composite reliability (CR) values for all constructs were above the conventional cutoff of 0.70, indicating satisfactory internal consistency. Furthermore, the average variance extracted (AVE) values exceeded 0.50 for all latent variables, supporting acceptable convergent validity. These results are presented in Table 2.

**Table 2:** Reliability and convergent validity for the measurement model.

Constructs	Items	Loadings	S.E.	CR	AVE
PS	PS1	0.826***		0.884	0.718
	PS2	0.827***	0.05		
	PS3	0.888***	0.047		
IM	IM1	0.870***		0.825	0.611
	IM2	0.905***	0.057		
	IM3	0.902***	0.047		
EM	EM1	0.812***		0.884	0.718
	EM2	0.761***	0.057		
	EM3	0.834***	0.047		
TA	TA1	0.839***		0.909	0.770
	TA2	0.946***	0.041		
	TA3	0.844***	0.039		
SE	SE1	0.861***		0.925	0.803
	SE2	0.919***	0.037		
	SE3	0.908***	0.035		
LS	S1	0.950***		0.960	0.888
	S2	0.955***	0.021		
	S3	0.922***	0.024		
LE	LE1	0.937***		0.877	0.955
	LE2	0.921***	0.027		
	LE3	0.951***	0.024		

PS, Peer support; IM, Internal Motivation; EM, External Motivation; TA, Test Anxiety; SE, Self-Efficacy; LS, Learning Satisfaction; LE, Learning Engagement. *p < 0.05, **p < 0.01, ***p < 0.001.

As presented in Table 3, all HTMT values among the latent constructs were below the conservative threshold of 0.85 (Henseler et al., 2015). This result indicates that the constructs are empirically distinct, with no substantial issues of multicollinearity or conceptual overlap.

**Table 3:** HTMT values for discriminant validity.

Variable	PS	IM	EM	TA	SE	LS	LE
PS							
IM	0.443						
EM	0.443	0.670					
TA	0.431	0.548	0.412				
SE	0.510	0.646	0.609	0.577			
LS	0.617	0.684	0.586	0.675	0.778		
LE	0.579	0.746	0.670	0.605	0.804	0.804	

PS, Peer support; IM, Internal Motivation; EM, External Motivation; TA, Test Anxiety; SE, Self-Efficacy; LS, Learning Satisfaction; LE, Learning Engagement.

### Structural model results

The proposed research model was established using structural equation modeling. The goodness-of-ft indices were well acceptable for the structural equation model (SEM): *X2* = 385.499, *df* = 167, *X2/df* = 2.31, CFI = 0.979, TLI = 0.974, RMSEA = 0.051, SRMR = 0.033. Table 4 reports the standardized path coefficients (*β*) for the hypothesized structural relationships in the proposed model. The strongest positive effect was observed from peer support to self-efficacy (*β* = 0.506, *p* < 0.001), while the weakest significant effect was found from peer support to learning engagement (*β* = 0.155, *p* < 0.001).

**Table 4:** Reliability and convergent validity for the measurement model.

Constructs	*β*	S.E.	C.R.	*z*-value	Results
PS → IM	0.458***	0.059	9.505	7.763	Supported
PS → EM	0.448***	0.057	8.667	7.860	Supported
PS → TA	−0.412***	0.065	−8.528	−6.338	Supported
PS → SE	0.506***	0.055	10.426	9.200	Supported
PS → LS	0.233***	0.049	6.694	4.755	Supported
PS → LE	0.155***	0.036	4.631	4.306	Supported
IM → LS	0.218***	0.054	4.652	4.037	Supported
EM → LS	0.019	0.059	0.412	0.322	Not Supported
TA → LS	−0.190***	0.036	−5.494	−2.778	Supported
SE → LS	0.399***	0.055	9.087	7.255	Supported
IM → LE	0.201***	0.040	4.435	5.025	Supported
EM → LE	0.175***	0.044	3.878	3.977	Supported
TA → LE	−0.031	0.026	−0.928	−1.192	Not Supported
SE → LE	0.480***	0.041	11.13	11.707	Supported

PS, Peer support; IM, Internal Motivation; EM, External Motivation; TA, Test Anxiety; SE, Self-Efficacy; LS, Learning Satisfaction; LE, Learning Engagement. *p < 0.05, **p < 0.01, ***p < 0.001.

The significance of the mediating effects was tested using a bootstrap method with 1,000 resamples to calculate the 95% confidence intervals (CIs). As shown in Table 5, internal motivation (*β* = 0.141, 95% CI [0.075, 0.245]), test anxiety (*β* = 0.111, 95% CI [0.049, 0.190]), and self-efficacy (*β* = 0.262, 95% CI [0.162, 0.420]) significantly mediated the relationship between peer support and learning satisfaction. Internal motivation (*β* = 0.099, 95% CI [0.047, 0.171]), external motivation (*β* = 0.085, 95% CI [0.035, 0.154]), and self-efficacy (*β* = 0.262, 95% CI [0.143, 0.386]) significantly mediated the relationship between peer support and learning engagement.

**Table 5:** Bootstrap analysis of the multiple mediating effects.

Constructs	Effect size	SE	95% CI Lower limit	95%CI Upper limit
PS → IM → LS	0.141**	0.042	0.075	0.245
PS → EM → LS	0.012	0.034	−0.055	0.084
PS → TA → LS	0.111**	0.035	0.049	0.190
PS → SE → LS	0.262**	0.068	0.162	0.420
PS → IM → LE	0.099**	0.031	0.047	0.171
PS → EM → LE	0.085**	0.030	0.035	0.154
PS → TA → LE	0.014	0.019	−0.017	0.057
PS → SE → LE	0.262**	0.062	0.143	0.386

PS, Peer support; IM, Internal Motivation; EM, External Motivation; TA, Test Anxiety; SE, Self-Efficacy; LS, Learning Satisfaction; LE, Learning Engagement. *p < 0.05, **p < 0.01, ***p < 0.001.

### Results of measurement invariance

This model exhibited an acceptable fit to the data (*X2/df* = 2.366, CFI = 0.978, RMSEA = 0.052, SRMR = 0.034), including the livestream group data (*X2/df* = 1.971, CFI = 0.962, RMSEA = 0.067, SRMR = 0.054) and the prerecorded video group data (*X2/df* = 1.705, CFI = 0.981, RMSEA = 0.049, SRMR = 0.037); in all cases, the goodness-of-fit indices were acceptable (Hair et al., 2009). The measurement weights, structural weights, and measurement residuals were constrained to test the stability of the models between the livestream group and the prerecorded video group (Table 6).

**Table 6:** Descriptive statistics and correlations.

Model	*X2/df*	CFI	RMSEA (90% CI)	SRMR	Model comp	∆CFI	Decision
All groups	2.366	0.978	0.052 [0.045, 0.058]	0.034	–	–	–
Livestreams	1.971	0.962	0.067 [0.056, 0.078]	0.054	–	–	–
Prerecorded videos	1.705	0.981	0.049 [0.039, 0.059]	0.037	–	–	–
M1: Unconstrained	1.626	0.979	0.035 [0.030, 0.040]	0.049	–	–	–
M2: Measurement weights	1.620	0.978	0.035 [0.030, 0.040]	0.048	M1	0.001	Accept
M3: Structural weights	1.699	0.974	0.037 [0.032, 0.042]	0.048	M2	0.004	Accept
M4: Measurement residuals	1.752	0.970	0.039 [0.034, 0.043]	0.068	M3	0.004	Accept

PS, Peer support; IM, Internal Motivation; EM, External Motivation; TA, Test Anxiety; SE, Self-Efficacy; LS, Learning Satisfaction; LE, Learning Engagement. *p < 0.05, **p < 0.01, ***p < 0.001.

To explore the differences in the SWM category paths, we used multiple groups in the SEM analysis (i.e., the livestream group and the prerecorded video group). The chi-square differences between the unconstrained and fully constrained models are shown in Table 7 and indicate that the two groups exhibited notable differences (∆*X2* = 21.625; *∆df* = 9; *p* = 0.01).

**Table 7:** Measurement invariance testing.

Overall model	*X2*	*df*	*p-value*	Invariant
Unconstrained model	634.523	350	–	–
Fully constrained model	673.280	364	–	–
Difference	38.757	14	0.000	NO

**Figure 2:**
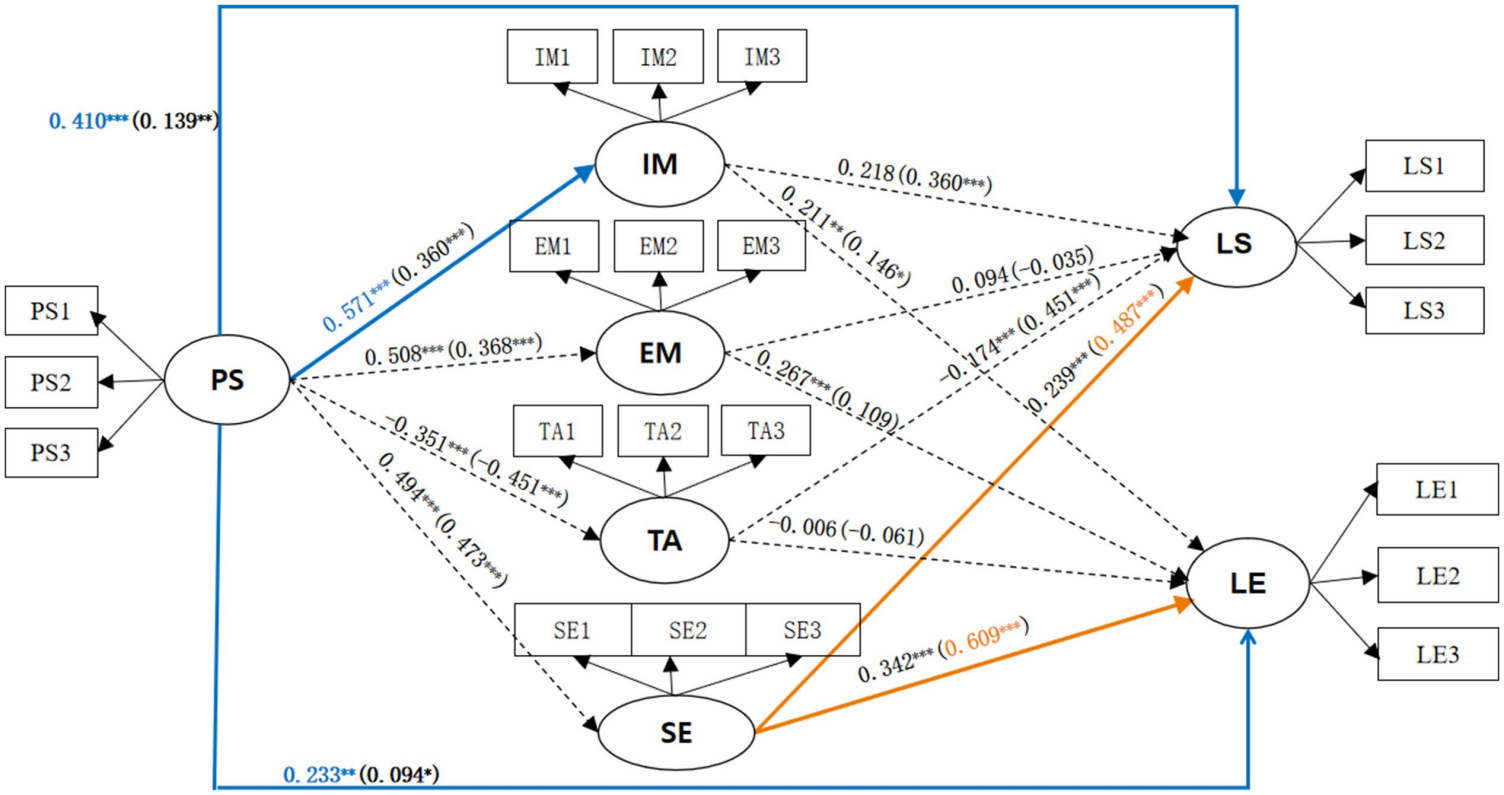
Mediation model for the livestream group (left) and the prerecorded video group (right) PS, Peer Support; IM, Internal Motivation; EM, External Motivation; TA, Test Anxiety; SE, Self-Efficacy; LS, Learning Satisfaction; LE, Learning Engagement. *p < 0.05, **p < 0.01, ***p < 0.001. Solid lines: indicate significant paths; While dotted lines: indicate non-significant paths. Blue lines and text correspond to the livestream group; Orange lines and text correspond to the prerecorded video group.

Furthermore, multigroup structural equation modeling (SEM) was conducted to assess whether structural path differences existed between students who used livestreams SWM and those who used prerecorded SWM videos. As shown in Table 8, the results indicated that five path coefficients significantly differed between the two groups. All reported path coefficients were statistically significant. Students in the livestream group exhibited significantly stronger path coefficients PS → IM (*β* = 0.571, *p* < 0.001), PS → LS (*β* = 0.410, *p* < 0.001), PS → LE (*β* = 0.233, *p* < 0.001) than those in the prerecorded group. Conversely, the path SE → LS (*β* = 0.487 *p* < 0.01) and the path SE → LE (*β* = 0.609, *p* < 0.01) were significantly stronger in the prerecorded video group compared to the livestream group. These differential path are visually presented in Figure 2.

**Table 8:** Direct and indirect effects test for the full sample, the livestream group and the prerecorded video group.

Path	Livestream Group			Prerecorded video Group			*∆X2 ∆df = 1*	*p*	Results
	*β*	S.E	*B*	*β*	S.E	*B*			
PS → IM	0.571***	0.103	0.809	0.360***	0.074	0.407	5.338	0.021	Supported
PS → EM	0.508***	0.094	0.600	0.368***	0.075	0.399	1.878	0.171	Reject
PS → TA	−0.351***	0.105	0.809	−0.451***	0.088	−0.618	1.388	0.239	Reject
PS → SE	0.571***	0.095	0.579	0.473***	0.072	0.529	0.264	0.607	Reject
PS → LS	0.410***	0.092	0.617	0.139**	0.061	0.195	12.174	<0.001	Supported
PS → LE	0.233***	0.066	0.270	0.094*	0.045	0.100	4.276	0.039	Supported
IM → LS	0.116	0.073	0.123	0.242***	0.083	0.300	2.104	0.147	Reject
EM → LS	0.094	0.090	0.119	−0.035	0.079	−0.046	−2.005	0.157	Reject
TA → LS	−0.174***	0.053	−0.186	−0.198***	0.051	−0.204	−0.422	0.178	Reject
SE → LS	0.289***	0.083	0.370	0.487***	0.072	0.613	4.984	0.026	Supported
IM → LE	0.211***	0.055	0.172	0.146*	0.061	0.137	0.343	0.558	Reject
EM → LE	0.267***	0.069	0.262	0.109	0.059	0.107	3.807	0.079	Reject
TA → LE	−0.006	0.039	−0.005	−0.061	0.037	−0.048	0.694	0.405	Reject
SE → LE	0.342***	0.039	0.005	0.609***	0.056	0.580	7.594	0.006	Supported

PS, Peer support; IM, Internal Motivation; EM, External Motivation; TA, Test Anxiety; SE, Self-Efficacy; LS, Learning Satisfaction; LE, Learning Engagement. *p < 0.05, **p < 0.01, ***p < 0.001.

## Discussion

The results support H1, indicating that peer support significantly and positively predicts intrinsic motivation (*β* = 0.458, *p* < 0.001), emotional motivation (*β* = 0.448, *p* < 0.001), self-efficacy (*β* = 0.506, *p* < 0.001), learning satisfaction (*β* = 0.233, *p* < 0.001), and learning engagement (*β* = 0.155, *p* < 0.001). In contrast, peer support negatively predicts test anxiety (*β* = −0.412, *p* < 0.001).

The findings support H2 and H5, intrinsic motivation significantly mediated the relationship between peer support and both learning satisfaction (*β* = 0.141, 95% CI [0.075, 0.245]) and learning engagement (*β* = 0.099, 95% CI [0.047, 0.171]). Self-efficacy mediated the relationship between peer support and both learning satisfaction (*β* = 0.262, 95% CI [0.162, 0.420]) and learning engagement (*β* = 0.262, 95% CI [0.143, 0.386]). Partial support was found for H3 and H4. Emotional motivation mediated the relationship between peer support and learning satisfaction (*β* = 0.085, 95% CI [0.035, 0.154]). Test anxiety mediated the relationship between peer support and learning satisfaction (*β* = 0.111, 95% CI [0.049, 0.190]).

The results support H8, specifically, students in the livestream group exhibited significantly stronger path coefficients from peer support to intrinsic motivation (*β* = 0.571, *p* < 0.001), peer support to learning satisfaction (*β* = 0.410, *p* < 0.001), and peer support to learning engagement (*β* = 0.233, *p* < 0.001) than those in the prerecorded group. Conversely, the path from self-efficacy to learning satisfaction (*β* = 0.487, *p* < 0.01) and the path from self-efficacy to learning engagement (*β* = 0.609, *p* < 0.01) were significantly stronger in the prerecorded video group compared to the livestream group.

### The impact of peer support

Peer support was a positive predictor of learning satisfaction and learning engagement, which is consistent with the findings of previous research that has shown that the higher the level of peer support is, the greater the students’ satisfaction and learning engagement (Lai et al., 2019). In SWM, learners use likes, bullet comments, live chats, comments, and other methods to facilitate rich emotional communication and information-sharing interactions, which can help alleviate their loneliness, improve their learning mindsets (Yunianto and Putridinanti, 2022) and increase their satisfaction. In addition, the diverse learning scenarios offered by visual symbols (such as students learning in a library), textual symbols (counting down to goals or time rankings in terms of students’ investment in learning), and sound symbols (such as white noise) in SWM provide companionship during learning and facilitate multiple strategies that can strengthen or optimize the learning path and increase learning engagement.

Peer support had significantly positive predictive effects on internal motivation, external motivation, and self-efficacy, a conclusion which is consistent with the findings of previous research (Fidan and Gencel, 2022). Learners who exhibit similar learning goals and emotional needs collaborate in the context of SWM, thereby establishing a virtual learning community with clear attributes (Wang et al., 2022). In this community, experiencing personalized learning styles by interacting with different learners increases the fun of learning and enhances participants’ internal motivation (Kim and Ryoo, 2023), and when learners aim to display an ideal image of “good students” to their peers to gain more recognition, this situation also promotes learners’ external motivation. In addition, supportive peer interactions help cultivate learners’ confidence in their ability to complete learning tasks (Jung and Choi, 2023).

Peer support has a significant negative predictive effect on test anxiety, which is consistent with the findings reported by Yang and Wang (2023), who showed that stronger peer support is associated with lower test anxiety. Sharing stress and emotions among peers can help individuals mitigate their anxiety (Hou et al., 2023). These findings not only emphasize the impact of peer support on learning outcomes in SWM but also highlight the importance of individual psychological factors in this context.

It is evident that learners who participate in SWM can also receive peer support in the real world; accordingly, why would they choose to seek companionship in cyberspace? The key to answering this question lies in the uniqueness of the peer support offered by SWM (Wang et al., 2022). Granovetter (1983) noted that relationships that occur in real time and in a shared physical space can become complex and tense, and adjusting to other people’s schedules and encountering other people’s emotions can represent “troubles” associated with offline partnerships. However, Internet can alleviate the burden of social relationships (Hjarvard, 2008). In SWM, this “light social relationships” relationships satisfies contemporary college students’ dual needs for social interaction and learning.

### The mediating effects of learning motivation, test anxiety, and self-efficacy

Internal motivation has a mediating effect on the relationships between peer support and both learning satisfaction and learning engagement. External motivation has a mediating effect on the relationship between peer support and learning engagement. Individuals enjoy learning and become interested in it as a result of communication and exchange among peers. Due to their increasing internal motivation, learners are becoming more willing to invest effort into learning, thus enhancing their learning satisfaction and learning engagement (Wei and Chou, 2020). Surprisingly, this study revealed that the stronger the external motivation is, the higher the level of learning engagement, which is inconsistent with the findings reported by Zaccone and Pedrini (2019), who indicated that external motivation negatively affects learning outcomes. This difference may be attributed to the social media era, where individuals tend to alter their behavior when they are aware of being observed (Cañigueral and Hamilton, 2019), this compels them to suppress the urge for entertainment and focus more intently on academic tasks, which in turn supports their goal achievement (Hou et al., 2023).

Test anxiety has a mediating effect on the relationship between peer support and satisfaction. Students who have access to more peer support have more positive views of themselves (Wentzel et al., 2017), when such students encounter academic setbacks, they can address those setbacks more effectively with a positive outlook and thus exhibit lower levels of exam anxiety (Lei et al., 2021). Students who experience relief from test anxiety can focus more effectively on their studies, encouraging from peers and maintaining mental optimism can improve satisfaction during the learning process (Yunianto and Putridinanti, 2022), which is consistent with the results of this study. Intense competition results in a continuous weakening of learners’ enthusiasm and confidence within educational settings. Long-term high-intensity learning can easily trigger fear of exam failure, peer companionship plays a key role in meeting emotional needs while promoting the sharing of exam tips and preparation strategies. These interactions help ease feelings of loneliness and reduce psychological stress, improving their overall learning experience.

Self-Efficacy has a mediating effect on the relationships between peer support and both learning satisfaction and learning engagement. Gan et al. (2023) reported that learners who engage in more peer interactions have greater self-efficacy, that students are willing to take on the challenges associated with learning difficulties with more enthusiasm. Handayani et al. (2021) also reported that the higher the level of peer support is, the greater students’ self-efficacy, learning satisfaction, and learning engagement (Sökmen, 2021). In SWM, Learners use modern media tools and learning technologies to share their study processes, visualizing their own and others’ learning activities across both temporal and visual dimensions, this allows learners to track study progress and quantify both their own and their peers’ efforts, helps learners adjust their learning habits (Wu and Zhang, 2021), to achieve more learning outcomes.

### Analysis of the differences between live streams and prerecorded videos

In SWM, on the path underlying the impact of peer support on internal motivation, learning satisfaction, and learning engagement, livestreams have greater effects than did prerecorded videos. Livestreams through establishing real-time linkages, immediate feedback and visible process, create a dynamic study environment. In this environment, each participant is both an learner and a co-creator of study environment, feeling part of an active and supportive community, which significantly contributes to their involvement in the learning interest. Additionally, with the global accessibility and scalability provided by internet, interactive community by continuously drawing new participants. It not only meet learners’ needs for peer emotions and interaction but also preserves the autonomy of learners, who are able to enter and leave at any time (Hong, 2023), reduces the psychological burden on social relationships (Wu and Zhang, 2021), satisfies the psychological needs of Generation Z college students. However, not all learners are willing to expose themselves in the context of live broadcasts. The discomfort associated with public exposure is not merely a concern about privacy leakage risks but also reflects differences in the degree to which different learners accept new technologies, and places higher requirements for learning scenarios and independence.

On the path underlying the impact of self-efficacy on learning satisfaction and learning engagement, prerecorded videos have greater effects than did livestreams. Students who choose based on their own learning pace and interest of prerecorded videos. Prerecorded videos have a high level of aesthetic quality, are often carefully edited and contain high-quality audiovisual information, audio elements and visual cues of the setting lead them to imagine themselves within the created environment (Li & Li, 2023), rich learning scenarios, and diverse learning strategies, effectively attracting the attention of learners. Prerecorded videos, through close-up shots of the video creator’s facial expressions or hand movements, learners repeatedly engage with content from the same creator, their growing familiarity with the video’s producer nurtures a profound, “one-to-one” sense of companionship, this familiarity and comfort with the content creator enhance the study experience.

The choice of livestreams and prerecorded videos by learners not only reflects differences in learning styles but also shows SWM affords different peer support to learners. Learners in livestreams collaboratively shape a dynamic collective learning environment, learners receive both encouragement and oversight from peers, draw strength and accountability from collective (Chu and Chu, 2010), enriching the collective learning experience. Prerecorded videos are particularly appealing to learners who prefer solitude and do not like external distractions. SWM videos creators provide both companionship and serve as a model of study emulation. The choice of livestreams and prerecorded videos by learners also reflects differences in learning styles and the different types of social support needed, satisfying the way of personalized learning lies in help learners’ identification of the most suitable learning methods for themselves.

## Conclusion

This study used theoretical framework of SCT and SPT construction to enhance our understanding of the digital study environments through SWM. This theoretical framework analyzes the pivotal role of peer support in online educational experiences and explains how the digital virtual presence of peers contributes to fulfilling learners’ needs for social interaction and emotional, thereby reducing feelings of isolation and enhancing overall learning outcomes. Moreover, the distinction in peer support between livestreams and prerecorded videos not only caters to personalized learning preferences but also shows how different types of peer interactions influence learners’ psychological states and learning effectiveness. This study used theoretical framework of SCT and SPT construction to enhance our understanding of the digital study environments through SWM. This theoretical framework analyzes the pivotal role of peer support in online educational experiences and explains how the digital virtual presence of peers contributes to fulfilling learners’ needs for social interaction and emotional, thereby reducing feelings of isolation and enhancing overall learning outcomes. Moreover, the distinction in peer support between livestreams and prerecorded videos not only caters to personalized learning preferences but also shows how different types of peer interactions influence learners’ psychological states and learning effectiveness. On a theoretical level, this study demonstrates the synergistic potential of SCT and SPT when combined in digital environments, this advancement contributes significantly on building supportive online self-directed learning environments. On a practical level, this study confirms the effectiveness of SWM on learning outcomes.

### Study limitations and future scope

This study employed a cross-sectional survey of Generation Z Chinese college students, study relied on a relatively small amount of data, so the findings are somewhat limited in terms of their applicability to learners from diverse educational backgrounds. Although key exogenous variables were accounted for, potential confounding effects cannot be entirely ruled out. Future studies may benefit from longitudinal or experimental designs and more diverse samples across age, education level, and cultural background. Such research would enhance understanding of SWM behaviors and inform the development of more personalized and effective digital learning environments.

## Data Availability

The raw data supporting the conclusions of this article will be made available by the authors without undue reservation.
